# Acceptability of Using Social Media Content in Mental Health Research: A Reflection. Comment on “Twitter Users’ Views on Mental Health Crisis Resolution Team Care Compared With Stakeholder Interviews and Focus Groups: Qualitative Analysis”

**DOI:** 10.2196/32475

**Published:** 2021-08-17

**Authors:** Nicola Morant, Natasha Chilman, Brynmor Lloyd-Evans, Jane Wackett, Sonia Johnson

**Affiliations:** 1 Division of Psychiatry University College London London United Kingdom; 2 Department of Psychological Medicine Institute of Psychiatry, Psychology & Neuroscience King's College London London United Kingdom; 3 Camden and Islington NHS Foundation Trust London United Kingdom

**Keywords:** Twitter, social media, qualitative, crisis resolution team, home treatment team, mental health, acute care, severe mental illness

Our recently published paper [1] that analyzed tweets about mental health crisis teams in the United Kingdom has sparked debate and some objections on social media, and we would like to clarify our position.

Our intention in conducting this research was to amplify voices and perspectives that may not be captured in more traditional qualitative research. Our findings highlight how, compared to views obtained using interviews and focus groups [2], Twitter users reported more negative experiences of mental health crisis services and described difficulties not identified by more standard qualitative methods. Social media research enables access to a broader range of voices, as it does not rely on recruitment via services. This has potential to usefully inform service developments, particularly in highlighting problems of access, engagement, or acceptability, and can help providers become more aware of how their services could be improved.

In planning this study, we obtained university ethics approval and followed guidance for social media research [3,4]. Only tweets from public accounts were included, and we did not record Twitter usernames or profiles. To avoid tweets being traceable to specific individuals, we paraphrased tweets rather than quoting them directly, using forms of words that were common across a body of similar material, and avoided using material about specific personal difficulties or circumstances.

Although we took care with our approach and felt we were able to reflect important concerns about a major service model, the publication of our paper has raised concerns that we feel other researchers should be aware of. The paper has divided opinion, with some social media users from a range of backgrounds strongly feeling that this form of social media data use is intrusive and that, even though material was in the public domain and was paraphrased, obtaining more specific consent was warranted. Conversations on Twitter may blur public and private, especially where it is used to share experiences among a community that includes people in vulnerable states.

Acceptability of research approaches, especially to people with relevant lived experience is clearly important. We would therefore not ourselves use a similar approach again without clear guidance being available that reflects what is acceptable to a fuller range of stakeholders. We recommend to other researchers being aware that use of material from public tweets may not be found acceptable even where ethical approval has been obtained and guidance followed. We hope this letter can contribute to the development of consensus and clear guidance on how researchers should navigate the complexity and variety of views that exist about social media content.
